# Moderate lifelong overexpression of tuberous sclerosis complex 1 (TSC1) improves health and survival in mice

**DOI:** 10.1038/s41598-017-00970-7

**Published:** 2017-04-11

**Authors:** Hong-Mei Zhang, Vivian Diaz, Michael E. Walsh, Yiqiang Zhang

**Affiliations:** 1 Department of Oncology, Xijing Hospital, Fourth Military Medical University, No. 169, Changle West Road, Xi’an, Shanxi 710032 P. R. China; 2 Greehey Children’s Cancer Research Institute, University of Texas Health Science Center at San Antonio, 7703 Floyd Curl Drive, San Antonio, Texas 78229 USA; 3 Sam and Ann Barshop Institute for Longevity and Aging Studies, University of Texas Health Science Center at San Antonio, 7703 Floyd Curl Drive, San Antonio, Texas 78229 USA; 4 Department of Cellular and Structural Biology, University of Texas Health Science Center at San Antonio, 7703 Floyd Curl Drive, San Antonio, Texas 78229 USA; 5 ETH Zurich, Department of Health Sciences and Technology LFW C 13.2, Universitätstrasse 2, 8092 Zurich, Switzerland

## Abstract

The tuberous sclerosis complex 1/2 (TSC1/2) is an endogenous regulator of the mechanistic target of rapamycin (mTOR). While mTOR has been shown to play an important role in health and aging, the role of TSC1/2 in aging has not been fully investigated. In the current study, a constitutive TSC1 transgenic (*Tsc1* ^*tg*^) mouse model was generated and characterized. mTORC1 signaling was reduced in majority of the tissues, except the brain. In contrast, mTORC2 signaling was enhanced in *Tsc1* ^*tg*^ mice. *Tsc1* ^*tg*^ mice are more tolerant to exhaustive exercises and less susceptible to isoproterenol-induced cardiac hypertrophy at both young and advanced ages. *Tsc1* ^*tg*^ mice have less fibrosis and inflammation in aged as well as isoproterenol-challenged heart than age-matched wild type mice. The female *Tsc1* ^*tg*^ mice exhibit a higher fat to lean mass ratio at advanced ages than age-matched wild type mice. More importantly, the lifespan increased significantly in female *Tsc1* ^*tg*^ mice, but not in male *Tsc1* ^*tg*^ mice. Collectively, our data demonstrated that moderate increase of TSC1 expression can enhance overall health, particularly cardiovascular health, and improve survival in a gender-specific manner.

## Introduction

mTOR is a central regulatory component for cell growth, proliferation, and cell size^1^. Through the interaction with a complex network of signaling pathways, mTOR regulates a wide spectrum of biological processes, including autophagy, protein translation, metabolism, appetite, energy, stress responses, and inflammatory response/immunity. The pivotal role of mTOR in health is reflected in the fact that deregulation of mTOR is associated with the development of many diseases, including inflammation, cancer, cardiac hypertrophy, and diabetes^2, 3^. Inhibition of mTOR signaling through either genetic or pharmaceutical approaches has been shown to slow aging in a variety of biological species^4–16^, indicating that mTOR is an important modulator of aging^17^.

mTOR exists in two complexes in mammalian cells, mTORC1 and mTORC2 (mTOR complex 1 and 2), with distinct roles in the regulation of biological processes^18, 19^. mTORC1, which is sensitive to the drug rapamycin, regulates ribosome biogenesis, protein translation and autophagy by phosphorylation of downstream targets, including S6K1, 4EBP1, and ULK1^20^. mTORC2, which is less sensitive to rapamycin, regulates the cytoskeleton and metabolism through the phosphorylation of a set of targets distinct from mTORC1, including AKT, PKCα, and SGK1.

mTORC1 is negatively regulated by a critical upstream inhibitor complex, the TSC1/2 complex^21^. Mutation of TSC1 or TSC2 leads to tuberous sclerosis, an autosomal-dominant genetic disease with the formation of benign tumors in multiple organ systems associated with hyperactivation of mTOR signaling^22^. TSC1/2 complex inhibits specifically mTORC1 by stimulating the conversion of a mTORC1 activator Rheb from an active form Rheb-GTP to an inactive form Rheb-GDP^23^. Interestingly, TSC1/2 has also been found to physically interact with and activate mTORC2, independent of Rheb^24^. TSC1/2 has been described as a molecular switchboard for multiple signals to activate or inhibit mTORC1 activity^21^, including growth hormone (insulin-IGF-Akt pathway), stresses (ERK/RSK), energy (the AMPK pathway), hypoxia (Redd1), and cytokines (IKKβ/NFκB). Because of its important role in mTOR signaling, animal models with TSC1/2 deletion or overexpression could be very useful for further understanding the role of mTOR signaling in different diseases and in aging. Previous study demonstrated that overexpression of TSC1 or TSC2 increased lifespan in Drosophila^12^. However, transgenic mammalian models of TSC1/2 have not been reported in aging studies, although a mouse model with muscle-specific overexpression of TSC1 or TSC2 has been reported to develop muscle atrophy, depending on the levels of transgene expression^25^. Besides its involvement in mTOR signaling, TSC1/2 has also been reported to function independently of mTOR^26^. It is therefore important to know the role of TSC1/2 in health and aging in both mTOR-dependent and –independent manner.

To help understand the role of TSC1/2 in health and aging, we initiated a study to establish a TSC1 transgenic mouse model, which was designed to overexpress a human TSC1 full length gene ubiquitously. Our study found that *Tsc1* ^*tg*^ mice exhibit alterations in multiple signaling pathways and biological processes that are associated with the mTOR-TSC1/2 axis, improvements in overall health, and specifically in females an improvement of survival.

## Results

### Generation of TSC1 transgenic mice

**Figure 1:**
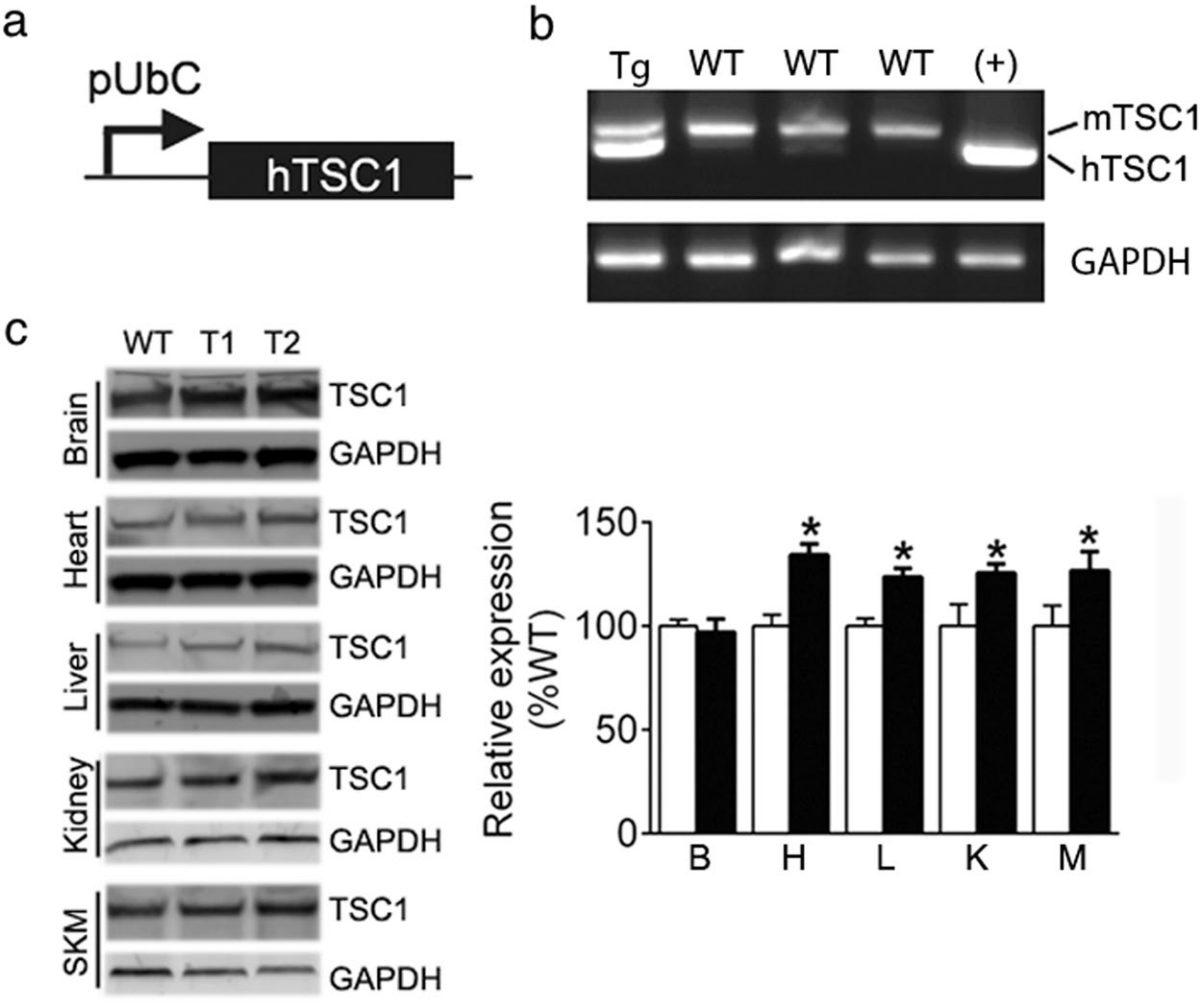
Generation of *Tsc1* ^*tg*^ mice. (**a**) Diagram of DNA construct used for generating hTSC1 transgenic mice. pUbC: ubiquitin C promoter. (**b**) Detection of hTSC1 transgene by PCR using genomic DNA isolated from the tail of one of the founder lines. Tg – hTSC1 transgenic founder #1; WT – wild type pups; (+) – positive control DNA for hTSC1. (**c**) Quantification of TSC1 protein expression in different tissues of *Tsc1* ^*tg*^ mice using western blot (n = 6 mice/group). B – brain; L – liver; H – heart; K – kidney; M – skeletal muscle. The asterisks indicate statistical significance between *Tsc1* ^*tg*^ and wild type mice (*p < 0.05).

Transgenic mice with hTSC1 expression were generated using the construct described in the materials and methods section (Fig. 1a). The integration of the transgene of hTSC1 was first confirmed in the founders and its offspring by detecting hTSC1 DNA using genomic DNA isolated from the tail of transgenic mice. As shown in Fig. 1b, the presence of the lower band which is specific for hTSC1 confirms the presence and transmission of hTSC1 gene in transgenic mice (the top band is the endogenous mouse TSC1). The expression of hTSC1 gene was subsequently confirmed by western blot analysis using TSC1 specific antibody. As shown in Fig. 1c, the expression of TSC1 protein increased approximately 30% in multiple tissues from *Tsc1* ^*tg*^ mice, including the heart, kidney, liver, and skeletal muscle. However, due to high levels of endogenous mouse TSC1 expression, the level of hTSC1 protein expression did not significantly altered the overall TSC1 protein pool in the brain of transgenic mice. Noticeably, the much higher level of endogenous TSC1 protein in adult mouse brain relative to other tissues is similar to what has been reported in human tissues^27^. The relatively small increase of TSC1 in the brain did not significantly affect downstream signaling and brain function as described in later sections of this report. The hTSC1 protein was still detectable in aged *Tsc1* ^*tg*^ mice (24–30 months of age) (liver and heart shown in, detected using a hTSC1-specific antibody), indicating a persistent expression of hTSC1 transgene in transgenic mice during aging.

### Phenotyping of *Tsc1*^*tg*^ mice

A high incidence of infant/newborn death was observed in *Tsc1* ^*tg*^ mice, with the survival rate of newborns around 50%. We suspect that the levels of hTSC1 transgene expression may be high in these groups of neonates, leading to the abnormally high neonatal death rate. We then performed quantitative real-time PCR analysis of hTSC1 transcription in the neonates. We found that the levels of hTSC1 mRNA were indeed significantly higher in a subpopulation of the dead neonates that were examined in this study (Figure S2A).

The breeding is successful and the litter size is not different from wild type mice (Supplemental Figure S2B). The average litter size is 4–5 for the following different breeding schemes: crossing wild male with transgenic female, or crossing transgenic male to wild type female, or crossing transgenic male to transgenic female mice. The average frequency of births is about one per month for all the aforementioned breeding schemes. The gender ratio of the offspring is normal (close to 1:1). When using homozygous male *Tsc1* ^*tg*^ mice to breed with wild type female mice or vice versa, the frequency of hemizygous *Tsc1* ^*tg*^ mice is close to 50%, indicating a normal Mendelian ratio in the transgene inheritance.

**Figure 2:**
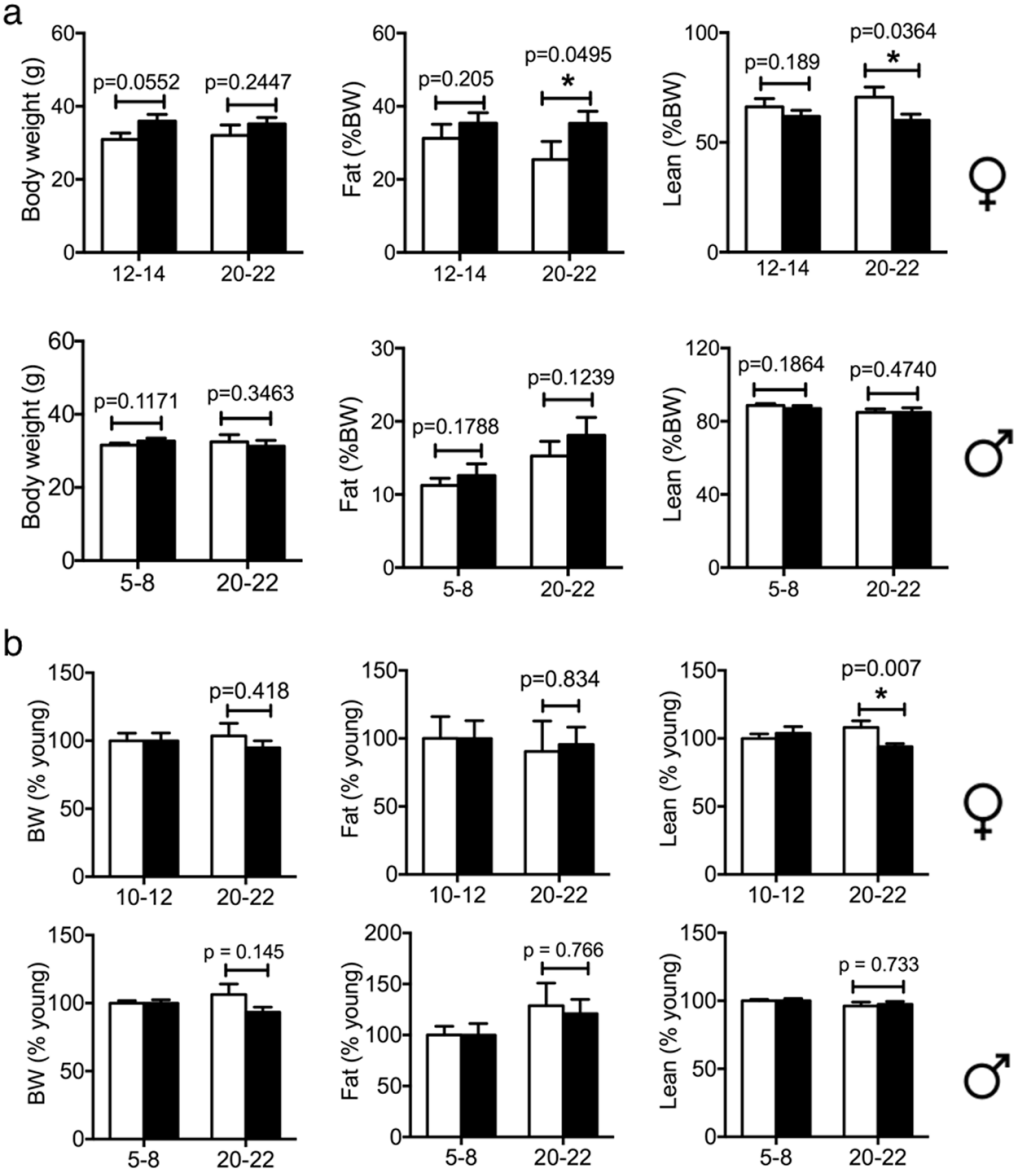
Body weight and composition. Body weight and composition of mice at different ages (specified in the figure) were determined using magnetic resonance imaging approach as described in the method section. (**a**) Body weight, lean and fat mass to body weight ratio. (**b**) Body weight, lean and fat mass normalized to young mice with the same gender and genotype. Male: 5–8 m mice, n = 17 for WT, n = 13 for *TSC1* ^*tg*^; 20–22 m mice, n = 18 for WT, n = 10 for *TSC1* ^*tg*^. Female: 12–14 m mice, n = 14 for WT, n = 15 for *TSC1* ^*tg*^; 20–22 m mice, n = 12 for WT, n = 19 for *TSC1* ^*tg*^. The asterisks indicate statistical significance between *Tsc1* ^*tg*^ and wild type mice (*p < 0.05).

The surviving *Tsc1* ^*tg*^ pups developed without obvious gross abnormality. The body weight and body composition was similar between wild type and *Tsc1* ^*tg*^ mice at younger ages in both sexes. However, female *Tsc1* ^*tg*^ mice had a small but significant reduction of lean body mass and increase of fat mass (as a percentage of body weight) at advanced ages (>20 months) than age-matched wild type control littermates (Fig. 2a). Male *Tsc1* ^*tg*^ mice, however, display no significant difference from age-matched wild type mice in body composition at any ages.

After normalizing the values against young mice, only significant age-associated changes between female *Tsc1* ^*tg*^ mice and wild type mice are the lean body mass ratio (Fig. 2b). The changes of fat mass and whole body weight from young to old mice were similar between both male and female *Tsc1* ^*tg*^ mice and wild type mice.

We further tested several behavioral and cognition parameters. No significant differences were detected between *Tsc1* ^*tg*^ mice and age-matched wild type mice in grip strength (Figure S2C), passive avoidance (Figure S2D), and motor neuron nerve conductivity (Figure S2E).

### *Tsc1*^*tg*^ mice have moderate but significant changes in multiple signaling pathways

**Figure 3:**
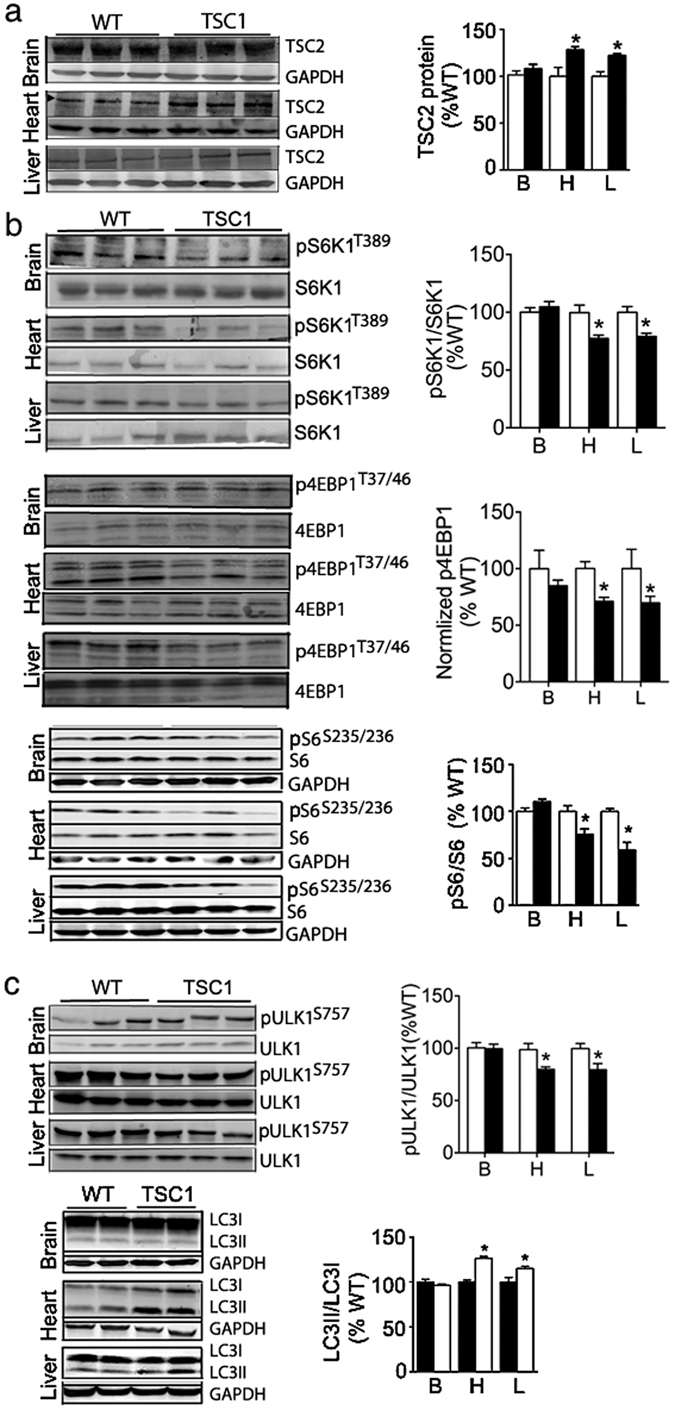
Effects of TSC1 overexpression on cell signaling. Proteins extracted from different tissues were subjected to western blot analysis as described in the methods section (n > = 6 mice at 4–6 month of age for each genotype, includes both male and female mice). (**a**) TSC2 protein expression in different tissues. (**b**) mTOR signaling was monitored as the level of phosphorylation of S6K1 (threonine 389), ULK1 (serine 757), 4EBP1 (threonine 37/46), and S6 (serine 235/236). (**c**) The detection of autophagy related changes: Phosphorylation of ULK1 (serine 757) and the conversion of an autophagy marker LC3 from LC3I to LC3II (LC3II/LC3I ratio). WT – wild type littermates; TSC1 - *Tsc1* ^*tg*^ mice. B – brain; L – liver; H – heart. The band intensity was normalized to wild type mice in each group. The asterisks indicate statistical significance between *Tsc1* ^*tg*^ and wild type mice in a specific tissue (*p < 0.05).

Because TSC1 functions as a complex with TSC2 to inhibit mTOR signaling by preventing the degradation of TSC2^28^, we first investigated whether overexpression of TSC1 leads to stabilization of TSC2 in *Tsc1* ^*tg*^ mice. As shown in Fig. 3a, the levels of TSC2 proteins were indeed higher in heart and liver from *Tsc1* ^*tg*^ mice than age-matched wild type control littermates, suggesting that overexpression of TSC1 was able to stabilize TSC2 in *Tsc1* ^*tg*^ mice. However, the level of TSC2 did not change significantly in the brain of *Tsc1* ^*tg*^ mice.

Next, mTORC1 signaling was investigated by quantifying the phosphorylation of three mTORC1 subunits, S6K1, ULK1 and 4EBP1, as well as ribosome subunit 6 (S6), which is a substrate for S6K1^29^. As shown in Fig. 3b and c, the levels of phosphorylation of S6K1(T389), ULK1(S757), 4EBP1(T37/46), and S6(S235/236) decreased moderately but significantly (15–30%) in different tissues from *Tsc1* ^*tg*^ mice, including the heart and liver. Other tissues, including the kidney, spleen and skeletal muscle also exhibit reduced phosphorylation in S6 (Figure S1A). Correlated to the reduction of mTORC1 signaling, the conversion of LC3 (LC3II/LC3I ratio), which is a marker of autophagy^30^, increased moderately but significantly in the heart, kidney, liver, skeletal muscle, and spleen, suggesting an enhancement of autophagy in these tissues (Figs 3c and S1B). In the brain, the mTORC1 signaling and the ratio of LC3II/LC3I were not significantly altered in *Tsc1* ^*tg*^ mice when compared to age-matched wild type mice.

**Figure 4:**
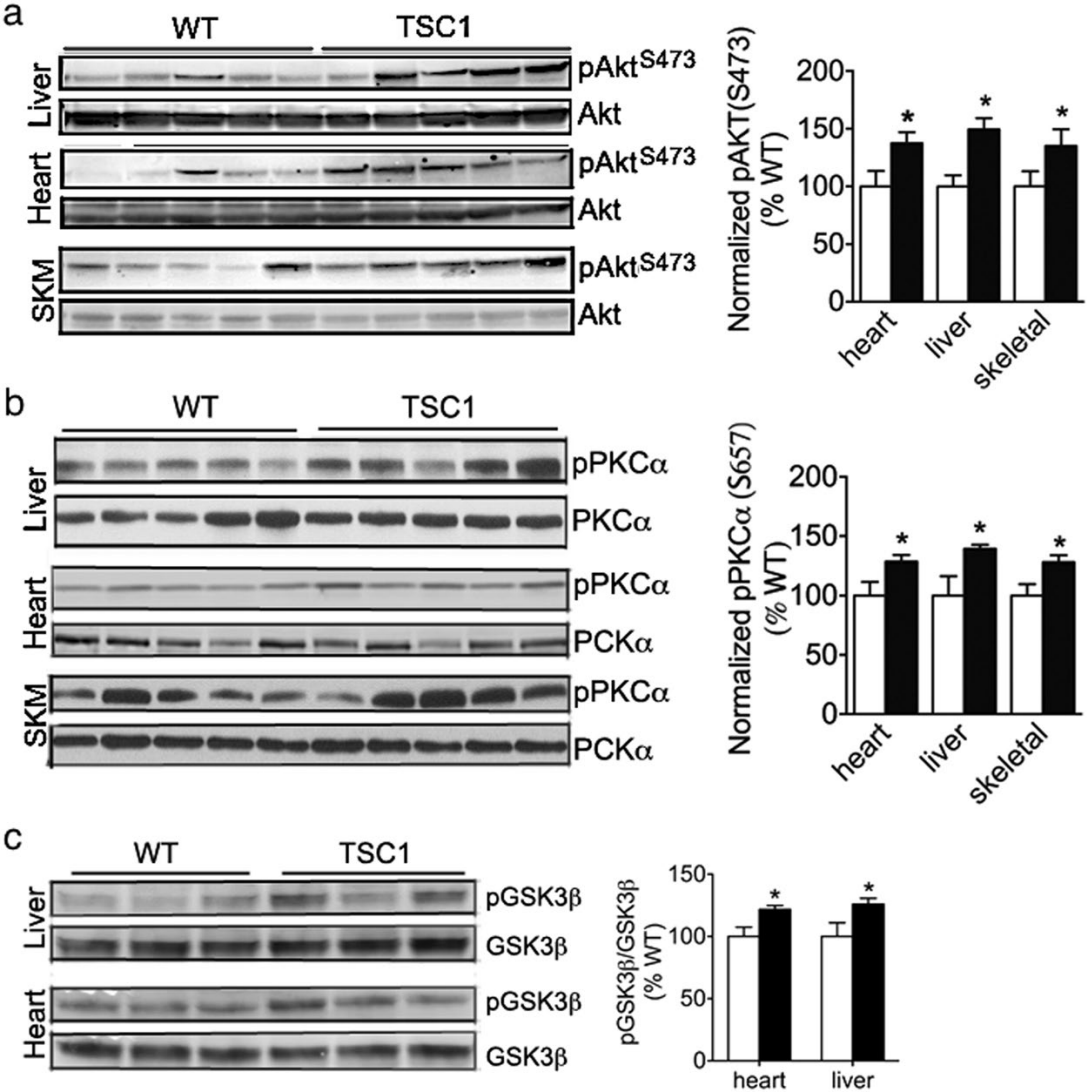
Changes of mTORC2 related signaling in *TSC1* ^*tg*^ mice. (**a**) Phosphorylation of ATK at serine 473 and (**b**) Phosphorylation of PCKα at serine 657 in the heart, liver and skeletal muscle (SKM). (**c**) Phosphorylation of GSK3β at serine 9 in liver and heart. The protein band intensity was normalized to wild type mice in each group. The asterisks indicate statistical significance between *Tsc1* ^*tg*^ and wild type mice (*p < 0.05, n = 6 for each group, includes both male and female mice).

While mTORC1 signaling was reduced, the signaling pathway associated with mTORC2 appears to be enhanced in *Tsc1* ^*tg*^ mice, indicated by the increases of the phosphorylation of AKT at serine 473 and PCKα at serine 657, which are two known substrates for mTORC2^31^. As shown in Fig. 4, the levels of phosphorylated AKT(S473) and PKCα(S657) increased moderately but significantly in the heart, liver, and skeletal muscle of *Tsc1* ^*tg*^ mice, suggesting an increase of mTORC2 activity. Interestingly, the phosphorylation of GSK3β at serine 9 was also increased (Fig. 4c). Because GSK3β (S9) phosphorylation is mediated by AKT, our data further support the activation of mTORC2 in *Tsc1* ^*tg*^ mice. However, the phosphorylation of ATK at threonine 308 was no significantly affected in *Tsc1* ^*tg*^ mice (Figure S1D).

### Food consumption, glucose tolerance and insulin sensitivity

Because mTOR signaling is associated with nutrition, energy metabolism and appetite, we next determined whether reduction of mTOR in *Tsc1* ^*tg*^ mice affected food consumption. Food consumption was measured for both male and female *Tsc1* ^*tg*^ mice at 4–6 months of age. As shown in Figure S3, both male and female *Tsc1* ^*tg*^ mice consumed similar amount of food per gram of body weight as that of age-matched wild type mice, indicating that a moderate reduction of mTOR signaling did not affect food consumption in young *Tsc1* ^*tg*^ mice. At advanced ages, both male and female *Tsc1* ^*tg*^ mice showed no alteration in the resting metabolic rate (RMR) and spontaneous activity (Supplemental Table S1) when measured at 18–20 months of age. However, during either light or dark cycle, the respiratory quotient (RQ) measured as the ratio between the volume of carbon oxide production and the volume of oxygen consumption in 18–20 month old female *Tsc1* ^*tg*^ mice is significantly higher than age-matched wild type littermates (Supplemental Table S1), suggesting female *Tsc1* ^*tg*^ mice preferentially utilize carbohydrate energy sources over fat. No difference was observed in male *Tsc1* ^*tg*^ mice.

**Figure 5:**
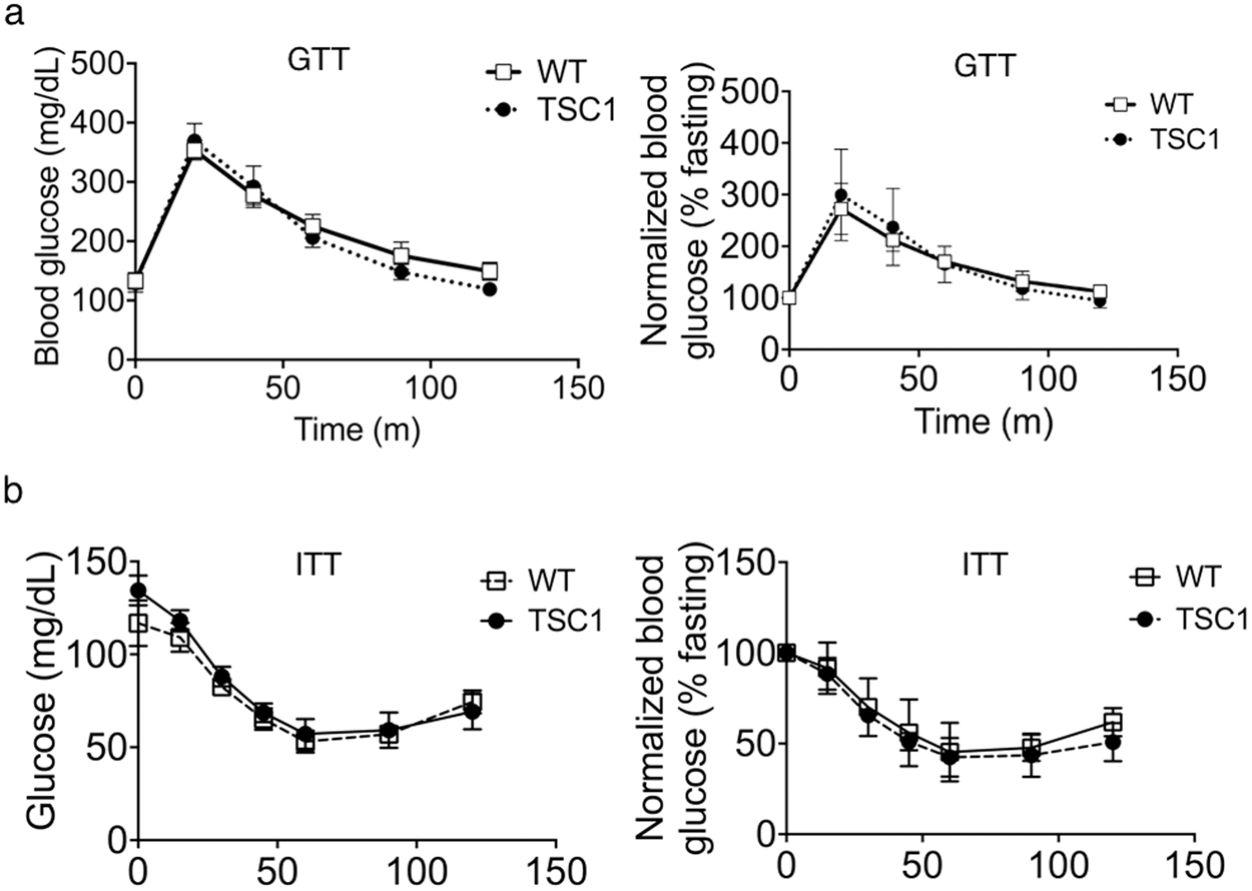
Glucose tolerance and insulin sensitivity in *TSC1* ^*tg*^ mice. (**a**) Glucose tolerance test (GTT) in 22–24 month old female mice (n = 6 mice in each group). (**b**) Insulin sensitivity test (ITT) in female 22–24 month old mice (n = 6 mice in each group). Blood glucose was measured every 15 or 30 minutes after either glucose or insulin injection. The left graphs corresponding to each graph on the left are plotted after normalizing to the levels of blood glucose just prior to the injection. WT – wild type mice (white square); TSC1 - *TSC1* ^*tg*^ mice (Solid black circle).

It has been reported previously that inhibition of mTOR signaling by rapamycin leads to glucose intolerance and insulin resistance^32–35^. We therefore compared the responses to glucose and insulin injection between *Tsc1* ^*tg*^ and wild type mice. First, the levels of blood glucose and insulin were measured. As shown in Figure S4A and S4B, the fasting blood glucose levels are not different between either young or old *Tsc1* ^*tg*^ mice and age-matched wild type mice. The plasma insulin level is also similar between female *Tsc1* ^*tg*^ mice and wild type mice under normal feeding conditions at both young and advanced ages (Figure S4C). We then compared the blood glucose clearance between *Tsc1* ^*tg*^ mice and age-matched wild type mice using GTT and ITT tests. As shown in Fig. 5a, female *Tsc1* ^*tg*^ mice at 22–24 months of age are similar in the clearance of blood glucose as age-matched wild type mice. Young *Tsc1* ^*tg*^ mice (4–6 month of age) also have similar responses to glucose injection as age-matched wild type mice (Figure S4D). Finally, *Tsc1* ^*tg*^ mice have similar insulin sensitivity as age-matched wild type mice at 22–24 months of age (Fig. 5b).

### *Tsc1*^*tg*^ mice are more tolerant to exhaustive exercise

**Figure 6:**
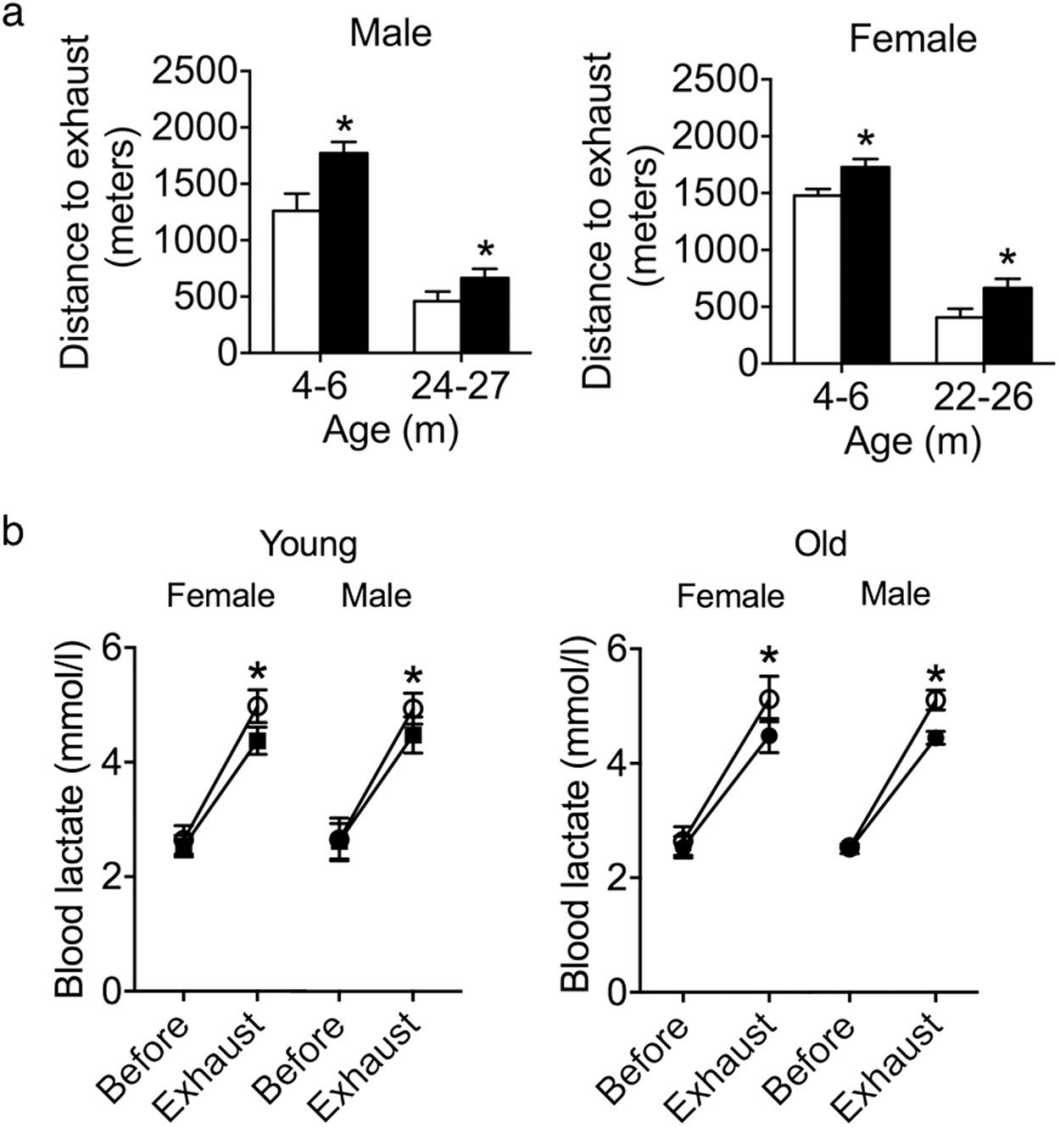
Exhaustive exercise on treadmill. (**a**) Running distance on treadmill till exhaustion for male and female mice at different ages (6–8, 24–27(female), 22–26 (male) months of age; n = 8 mice for each group). (**b**) Blood lactate levels before and after treadmill running test (n = 8 mice for each group). The asterisks indicate statistical significance between *Tsc1* ^*tg*^ (black solid bar or circle) and wild type mice (white blank bar or circle) (*P < 0.05). WT – wild type mice; TSC1 - *Tsc1* ^*tg*^ mice.

mTOR signaling is associated with energy metabolism, hence animal physical activity and strength^36^. One of the aging manifestations is the decline of physical activity and strength with age^37^. In this study, we investigated the tolerance of *Tsc1* ^*tg*^ mice to the treadmill exhaustive exercise running test at different ages. As shown in Fig. 6a, both male and female *Tsc1* ^*tg*^ mice have a statistically significant increase in treadmill running capacity at both young and old ages (4–6 and 22–27 months, respectively) compared to age-matched wild type littermates. *Tsc1* ^*tg*^ mice reached exhaustion and collapsed significantly later than age-matched wild type control mice. Blood lactate level was measured before and after treadmill running. The increase of blood lactate level in *Tsc1* ^*tg*^ mice after exercise is significantly less than that of wild type control mice (Fig. 6b).

### *Tsc1*^*tg*^ mice are more resistant to cardiac hypertrophy

**Figure 7:**
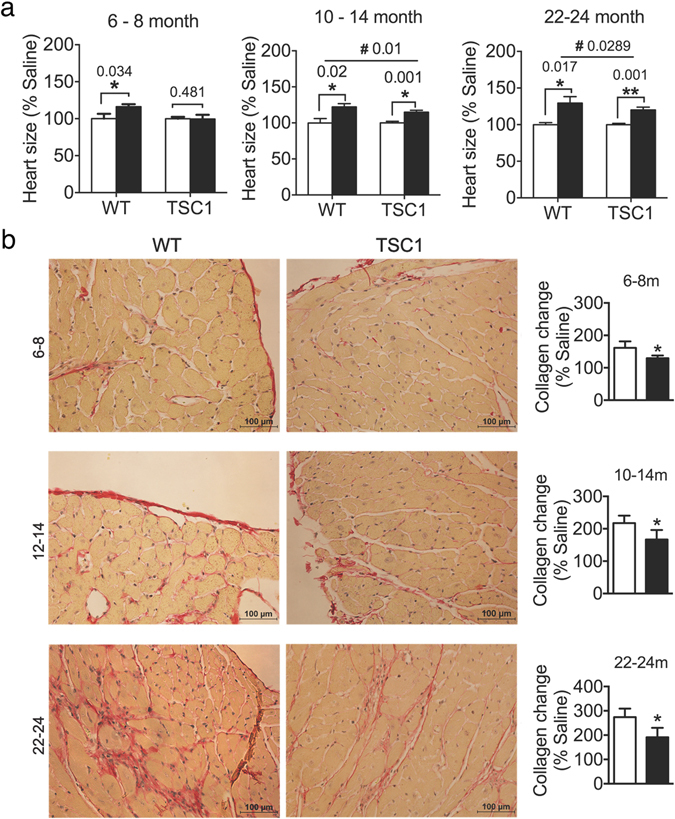
Cardiac function analysis after isoproterenol injection. (**a**) Heart size (ratio to body weight) in young (6–8 month, n = 8 for each group), middle aged (10–14 months, n = 8 WT, n = 12 *TSC1* ^*tg*^), and old (22–24 months, n = 13 WT, n = 15 *TSC1* ^*tg*^) female mice before and after isoproterenol challenge. White bar – mice with saline; black bar – mice with isoproterenol injection. Asterisks (*p < 0.05 and **p < 0.01) indicate statistical significance between isoproterenol injected mice and saline injected mice. Ampersand (^#^p < 0.05) indicates statistical significance in the degree of hypertrophy after isoproterenol injection between *Tsc1* ^*tg*^ mice and wild type mice. (**b**) Histological examination of left cardiac tissue from young (6–8 month), middle aged (12–14 month), and old (22–24 month) female mice using Picrosirius Red Staining as described in the method section (n = 6 mice in each group). Collagen content changes during hypertrophy were calculated as a percentage of collagen content after isoproterenol against saline injected mice of the same genotype. Asterisks (*p < 0.05) indicate statistical significance between *Tsc1* ^*tg*^ mice and wild type mice.

Down regulation of mTOR signaling by rapamycin has been shown to reduce the development of cardiovascular diseases such as cardiac hypertrophy^38–45^. We therefore tested whether increased expression of TSC1 could affect cardiac function in a similar manner. Without any challenge, the cardiac function is similar between *Tsc1* ^*tg*^ mice and age-matched wild type littermates (data obtained from mice at 6–8 months of age, Supplemental S2). However, after challenged with isoproterenol, a compound used to induce pathological cardiac hypertrophy^46^, *Tsc1* ^*tg*^ mice developed significantly less cardiac hypertrophy with better preservation of cardiac function than age-matched wild type littermates. As shown in Fig. 7a, after isoproterenol injection, female *Tsc1* ^*tg*^ mice exhibited significantly less increases in heart size than age-matched wild type littermates at both young (6–8 month), middle (10–14 month), and advanced ages (22–24 month). Similar results were also observed in male *Tsc1* ^*tg*^ mice. The ejection fraction is significantly smaller in *Tsc1* ^*tg*^ mice than in wild type littermates as measured post isoproterenol challenge (data obtained from mice of 6–8 months of age were shown) (Figure S5).

Histological examination revealed that *TSC1* ^*tg*^ mice had significantly less fibrosis and collagen deposition in the heart after challenged with isoproterenol. As shown in Fig. 7b, the appearance of collagen fibers in the heart of female *TSC1* ^*tg*^ mice after ISO challenge increased significantly less than that of wild type control mice in all three age groups: young (6–8 months of age), middle (10–14 months of age), and old (22–24 months of age). The changes of collagen content after ISO challenge vs. saline control in each age group was quantified, as shown in the right of the histological staining images, to reflect the quantitative difference between *TSC1* ^*tg*^ mice and wild type control mice across different ages. These data further suggest that moderate TSC1 overexpression is beneficial to the heart against pathological hypertrophic stimulation.

**Figure 8:**
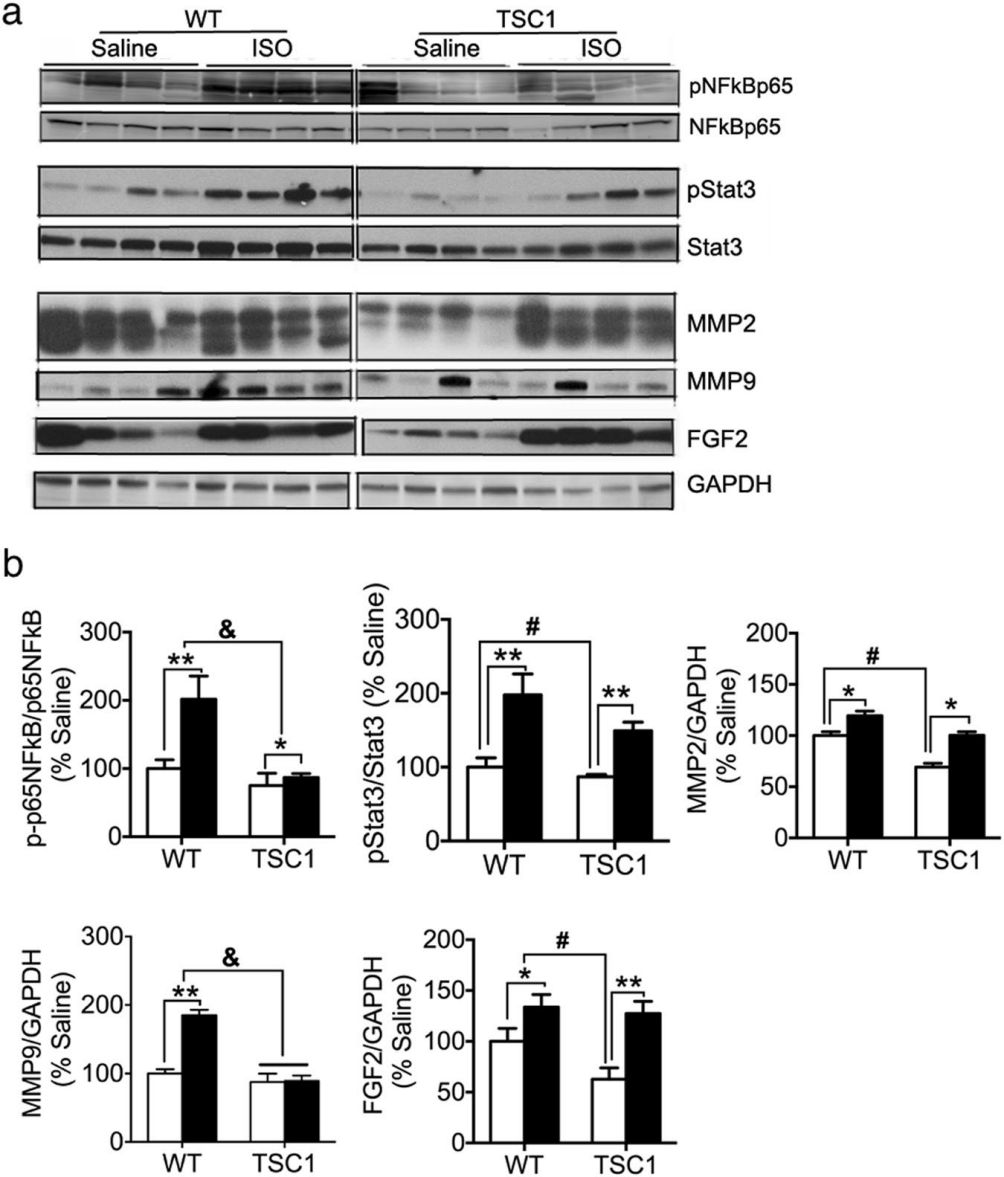
Fibrosis and inflammatory response in the heart of aged mice and the heart of mice after isoproterenol challenge. (**a**) Western blot analysis of NFκB, Stat3, FGF2 and MMP2/9 in heart protein extracts from old female mice (22–24 months of age, n = 6). (**b**) Quantification of protein band intensity (n = 6). WT – wild type mice; TSC1 - *Tsc1* ^*tg*^ mice. White bar – saline-injected mice; black bar – isoproterenol-injected mice. Asterisks (*p < 0.05 and **p < 0.01) indicate statistical significance between isoproterenol injected and saline injected mice of the same genotype as indicated in the graph.

Several signaling pathways involved in fibrosis and inflammation in the heart underwent changes in patterns that are significantly different between *Tsc1* ^*tg*^ mice and wild type mice at advanced ages and after ISO challenge. As shown in Fig. 8, two extracellular matrix metalloproteinases involved in cardiac remodeling, MMP2 and MMP9^47^, exhibit different patterns of changes between *Tsc1* ^*tg*^ and wild type mice at 22–24 months of age. The levels of cardiac MMP2 in aged wild type mice are significantly higher than that in *Tsc1* ^*tg*^ mice, while the levels of MMP9 remain similar between wild type and *Tsc1* ^*tg*^ mice. After ISO challenge, MMP2 increased significantly in both wild type and *Tsc1* ^*tg*^ mice, while MMP9 increased only significantly in wild type mice. In addition, FGF2 (Fibroblast Growth Factor 2), a growth factor involved in cardiac remodeling^48^, is expressed in significantly higher levels in aged wild type mice than *Tsc1* ^*tg*^ mice. After ISO injection, FGF2 increased further in wild type mice and also increased significantly in *Tsc1* ^*tg*^ mice. Finally, two pathways that are involved in inflammatory responses, the NFkB and Stat3 signaling pathways^49^, were less activated in the hearts of aged as well as ISO-challenged *Tsc1* ^*tg*^ mice than that in wild type mice, indicated by the lower levels of phosphorylation of NFkBp65 and Stat3. Our data therefore suggest that the hearts of aged as well as ISO-challenged *Tsc1* ^*tg*^ mice have less fibrosis and inflammation than wild type mice.

### Survival of *TSC1*^*tg*^ mice

**Figure 9:**
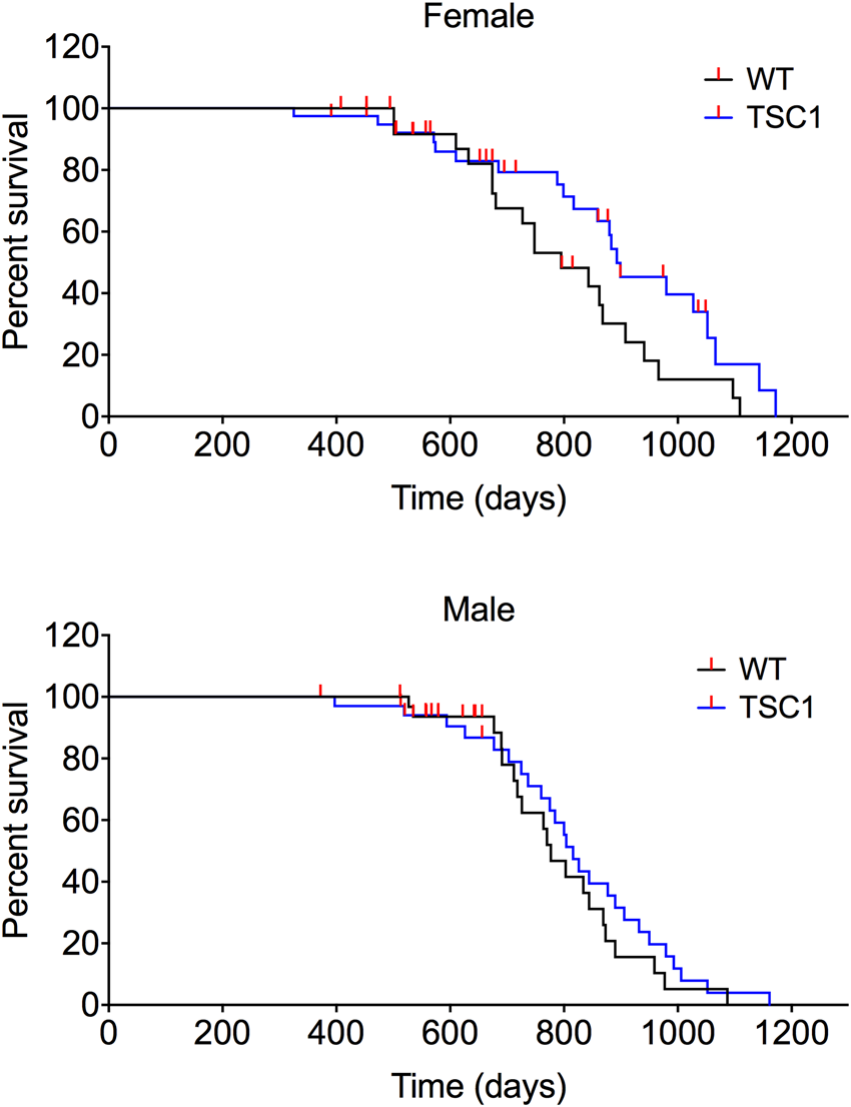
Lifespan analysis of *TSC1* ^*tg*^ mice. Survival curves are shown for female and male mice separately (67 females: 27 and 40 for wild type and *TSC1* ^*tg*^ mice separately; 67 males: 32 and 35 for wild type and *TSC1* ^*tg*^ mice respectively). The survival curve for female mice is significant, while not for male mice (p = 0.0282 for female mice; p = 0.195 for male mice, determined by one sided Log-rank test analysis).

A survival study was conducted using both male and female mice. As shown in Fig. 9 and Table S2, the survival is different between female and male *TSC1* ^*tg*^ mice. The lifespan of female *TSC1* ^*tg*^ mice is extended significantly (p = 0.028, one-sided Log-rank test), with an increase of 12.3% in median lifespan (893 vs. 795 days for *TSC1* ^*tg*^ mice and wild type mice, respectively). However, the survival of male *TSC1* ^*tg*^ mice is not significantly different from that of wild type mice (p = 0.195, one-sided Log-rank test), indicating a sex difference in the effect of TSC1 overexpression on lifespan extension.

## Discussion

In the current study we developed and characterized for the first time a mouse model that overexpresses TSC1 ubiquitously. Our data demonstrated that: (1) TSC1 overexpression did not lead to adverse health consequences to mice; (2) In fact, *Tsc1* ^*tg*^ mice are more tolerant to exhaustive exercises and more resistant to cardiac hypertrophic challenge during aging than wild littermates; (3) the survival of female *Tsc1* ^*tg*^ mice is significantly improved. These data suggest that TSC1 transgenic animal models could serve as new tools to understand the role of the TSC1/2 complex (mTOR related vs. non-mTOR related roles) in aging and age-associated diseases *in vivo*.

TSC1/2 is an important upstream regulator of mTOR signaling, which is a molecular switchboard for mTOR to respond to multiple signals^21^. Deregulated mTOR signaling has been implicated in major diseases, including cancer, metabolic disorders, neurological diseases, and inflammation^3^. Inhibition of mTOR using either genetic or pharmaceutical approaches has been found to extend lifespan in a wide spectrum of species from yeast to mammals^6–16, 50^. Previously, it has been shown that overexpression of TSC1 or TSC2 extended lifespan in *Drosophila* and improved cardiac function^12^. Our study using mouse model provide new evidence to support the notion that intervention in the gene expression of TSC1/2 complex can alter animal survival and health.

The observed gender differences in lifespan extension in *Tsc1* ^*tg*^ mice is not surprising. In fact, it is a common phenomenon observed in many genetic and pharmacological models^51^. Such gender differences in lifespan extension by mTOR inhibition have been reported in different animal models. Deletion of S6K1 in mice, a downstream target of mTORC1, led to significant increase of lifespan in female but not male mice^16^. Heterozygous deletion of both mTOR and mLST8 in mice led to lifespan extension in female but not male mice^33^. Pharmacological inhibition of mTOR in mice using rapamycin revealed that rapamycin has greater effect on female lifespan than on male lifespan^6, 7, 9^. It will be important to understand the mechanisms underlying this gender differences in lifespan extension by the intervention of mTOR signaling.

Although earlier studies using continuous rapamycin administration had some unexpected negative effects on mice, rats or human cells, including glucose intolerance^34, 35^ and insulin resistance^32, 33^, we did not observe changes in glucose homeostasis and metabolism in *Tsc1* ^*tg*^ mice, which is consistent with several recent studies. Wu *et al*.^10^ demonstrated that reduced mTOR expression in mice with mTOR hypomorphic alleles did not affect glucose tolerance, overall metabolic rate and energy expenditure. Lamming’s group recently demonstrated that intermittent rapamycin feeding did not alter glucose homeostasis^52^. Bartke’s group reported that the metabolic changes in rapamycin-fed mice was dependent on the duration of rapamycin treatment^53^. Insulin resistance and glucose intolerance occurred at short term rapamycin treatment (2 weeks), while insulin resistance disappeared after long term treatment (20 weeks).

Mice with rapamycin administration has also been reported to develop testicular degeneration and cataracts^50^. However, the male *Tsc1* ^*tg*^ mice are fertile with similar breeding capacity as wild type mice, indicating no testicular degeneration in male *Tsc1* ^*tg*^ mice. Interestingly, there is a noticeable difference in the survival rate of newborns between male *Tsc1* ^*tg*^ mice and wild type mice. The neonatal death is more than 50%, which is significantly higher comparing to wild type mice with less than 30% neonatal death rate. We speculate that at least two causes led to this abnormal neonatal death rate in *Tsc1* ^*tg*^ mice. First, the TSC1 transgene expression may be much higher in some embryos during development that leads to greater suppression of mTOR signaling. Because mTOR is critical to animal development^31^, the greater level of suppression of mTORC1 in the embryos may eventually lead to their demise. Second, the suppression of mTORC1 signaling may affect the mother’s nursing function (e.g., lack of enough milk for all the pups, etc.), which leads to the death of some pups. Some studies have linked mTOR signaling to milk production and lactation^54, 55^. Our data suggest that higher levels of hTSC1 expression during development may be one of the causes of the high neonatal death rate. Therefore, a different TSC1 transgenic animal model may serve the purpose better, such as an inducible model that expresses TSC1 or TSC2 transgene in a timely controlled manner to overcome its negative effect on development.

A previous study using rapamycin in C57BL/6 mice showed that male mice fed rapamycin experienced a reduction in whole body mass initially (from 19 to 25 months), while female mice did not^9^. Neither male nor female *Tsc1* ^*tg*^ mice lose weight in our study. Interestingly, female *Tsc1* ^*tg*^ mice have altered body composition with increased fat mass and decreased lean body mass (ratio to whole body mass) at advanced ages. The increase of fat deposit has been reported by Liu *et al*.^56^ in rapamycin- and high fat-fed male mice. However, Liu’s study did not report the effect of rapamycin on fat deposit in female mice. The cause of the gender difference in body composition in *Tsc1* ^*tg*^ mice is unknown. It may be due to the intrinsic gender differences of mTOR signaling in different tissues with age^57^. The change in body composition in female *Tsc1* ^*tg*^ mice is probably not due to food intake as being reported in mice fed rapamycin^9^, because we did not observe a significant difference in food consumption between *Tsc1* ^*tg*^ mice and wild type mice. The alteration of the ratio between fat mass and lean body mass in female *Tsc1* ^*tg*^ mice could be due to the changes of metabolism in fat usage and deposit. We further hypothesize that sex hormones may be involved in the differences between female and male *Tsc1* ^*tg*^ mice. Nonetheless, the underlying mechanisms by which overexpression of TSC1 leads to more fat mass in female *Tsc1* ^*tg*^ mice remains to be answered. It is worth to point out that the small gender differences did not affect the physiological outcomes tested in this study, including grip strength, motor neuron function, and cognition.

Although mTOR signaling plays an important role in cardiac development and function^58^, the heart appears to develop normally in *Tsc1* ^*tg*^ mice. Reduction of mTOR signaling has been shown to be beneficial to cardiac health and aging, especially cardiac hypertrophy^41–43, 59, 60^. *Tsc1* ^*tg*^ mice have increased resistance to cardiac hypertrophy challenge and are more resistant to exhaustive exercises, suggesting an improved cardiac function/health in *Tsc1* ^*tg*^ mice. *Tsc1* ^*tg*^ mice developed less fibrosis and inflammation in the heart during aging and when challenged with ISO, suggesting moderate TSC1 overexpression in our study improves cardiac health during aging. Our data further indicate that modulation of inflammation by TSC1 may play a very important role in aging and in cardiac response to hypertrophic stimulation. However, mTOR signaling has also been shown to be required for the protection of heart during ischemia/reperfusion (I/R) injury^61, 62^. Therefore, it is important to test whether reduction of mTOR signaling chronically by either genetic approaches or pharmaceutical approached will negatively affect the cardiac response to I/R injury during aging.

Although it has been shown to primarily affect the activation of mTORC1, TSC1/2 complex has also been reported to be required for proper activation of mTORC2^63^. The present study observed a small but significant increase of the phosphorylation of two mTORC2 substrates, AKT at serine 473 and PCKα at serine 657, suggesting the enhancement of mTORC2 signaling in *Tsc1* ^*tg*^ mice. The elevation of mTORC2 activity in *Tsc1* ^*tg*^ mice may partially explain the resistance of *Tsc1* ^*tg*^ mice to cardiac stresses and exhaustive exercises, which is in agreement with a recent study demonstrating that mTORC2 is required for normal cardiac function^64^.

It is worth to note that mTOR signaling was not altered in the brain of *Tsc1* ^*tg*^ mice. This is due to the expression of high levels of endogenous TSC1 in the brain tissue, which wipes out the effect of exogenous TSC1 on mTOR signaling in the brain. This could be the primary reason that we did not observe any significant changes in neurological tests. This problem can be addressed using brain tissue specific overexpression of TSC1/2 with a strong promoter. Although it is unfortunate that we did not observe neurological changes in *Tsc1* ^*tg*^ mice, this study still proves an important point that reduction of mTOR signaling in tissues other than the brain could still improve health and lifespan.

Finally, TSC1/2 complex has been found to function independently of mTOR^26^. Therefore, it is likely that the mTOR-independent function of TSC1/2 may also contribute to the lifespan extension and health improvement in *Tsc1* ^*tg*^ mice. It will be of great interest to identify those mTOR-independent functions of TSC1/2 that may be involved in health and aging in future studies.

In conclusion, our study has demonstrated that overexpression of TSC1 could extend lifespan and lead to better health in mice, particularly cardiac health.

## Materials and Methods

### Animals

All mice were fed a standard NIH-31 chow (Teklad Diet LM485; Harlan Teklad, Madison, WI) ad libitum and maintained under barrier conditions in micro-isolator cages on a 12-h dark/light cycle. For tissue collection, animals were sacrificed by CO2 inhalation followed by cervical dislocation, and the tissues were immediately excised and placed in liquid nitrogen.

All the animal procedures performed confirm to NIH guidelines and were approved by the institute animal care and use committee (IACUC) of the University of Texas Health Science Center at San Antonio, San Antonio, Texas.

### Generation of hTSC1 transgenic mice

Full-length hTSC1 cDNA (a kind gift from Dr. Kun-Liang Guan from the University of California at San Diego) was restriction digested and ligated into the mammalian expression vector pUB6-His (Invitrogen, CA) for ubiquitous expression under the control of ubiquitin C promoter (Fig. 1a). After the construct was confirmed by restriction digestion and DNA sequencing, the expression of hTSC1 protein was further determined and confirmed by transfecting *in vitro* tissue cultures. Transgenic mice were subsequently generated using this construct in the Transgenic Animal Model Core facility in University of Michigan. The genotypes of all offspring were determined by PCR amplification of mouse tail DNA using specific primers: 5′-TCAGTGTTAGACTAGTAAATTG-3′, and 5′-GCTGTTTCCCAGACTGTG-3′ for 35 cycles (94 °C 15 seconds, 52 °C 30 seconds, and 72 °C 50 seconds). Expression of hTSC1 in transgenic mice was determined at both transcription and protein levels using reverse transcriptional polymerase chain reaction (RT-PCR) with specific primers and western blotting analysis with TSC1 specific antibody respectively.

### Body composition measurement

Body composition and food consumption of transgenic mice and wild type littermates was determined as described elsewhere^65^. Briefly, body composition was measured using quantitative Magnetic Resonance Imaging (EchoMRI 3-in-1 System, Houston, TX), following the manufacturer’s recommendations.

### Western blot analysis

Protein expression and phosphorylation was determined by western blot analysis. Briefly, tissues were homogenized in ice-cold homogenization buffer (50 mM Tris-HCl, pH7.5, 0.5% Triton X-100) with the addition of cocktails of protease inhibitors and phosphatase inhibitors (Roche, Indianapolis, IN), followed by centrifugation at 10,000 g for 20 minutes at 4 °C. The supernatant was transferred to a new tube and protein concentration was determined using Bio-Rad protein assay reagents (Bio-Rad, CA). Equal amount of protein was separated by SDS-PAGE gel electrophoresis and transferred to nitrocellulose membrane. Individual protein was detected with species specific primary antibodies, followed by species specific secondary antibody conjugated with horse radish peroxidase (HRP). Primary antibodies used in this study are specific for: TSC1 (Cat. #4906), TSC2 (Cat. #3612), pTSC2(T1462) (Cat. #3611), S6 (Cat. #2217), pS6(Ser235/236) (Cat. #4856), 4EBP1 (Cat. #9452), p4EBP1(Thr37/46) (Cat. #9459), S6K1 (Cat. #9202), pS6K1(Thr389) (Cat. #9205), Akt (Cat. #9272), pAkt(Ser473) (Cat. #4060), pAkt(Thr308) (Cat. #9275), GSK3β (Cat. #9315), pGSK3β(Ser9) (Cat. #9336), ULK1 (Cat. #8054), Phospho-ULK1 (Ser757) (Cat. #6888), and LC3 (Cat. #2775) (all from Cell Signaling, Beverly, MA); hTSC1 (Epitomics, Cat. #1744-1; Cell Signaling, Cat. #3635); PKCα (Cat. ab32376), pPCKα(Ser657) (Cat. ab180848) (from Abcam, Cambridge, MA); GAPDH (from Santa Cruz Biotech., Dallas, Texas). The band signal was captured by a phosphor-imager (Typhoon scanner, GE, Piscataway, NJ) and quantified by the Image-Quant software (GE, Piscataway, NJ).

### Glucose tolerance test (GTT) and insulin tolerance test (ITT)

GTT were performed in mice (6 mice in each group) fasted overnight. The mice were weighed next morning and the dose of glucose were calculated for each mouse. Blood glucose was measured before the injection as baseline fasting blood glucose level. Glucose (20% solution in saline) was injected intraperitoneally at a dose of 1.5 mg kg^−1^ body weight. Blood glucose levels were measured using hand-held glucometer (One Touch Ultra) every 15 minutes post glucose injection.

Insulin tolerance test was conducted in mice (6 mice in each group) after 6 hours fasting. Insulin (0.25 IU solution in saline) was administered intraperitoneally at a dose of 0.75 IU/g body weight. Blood glucose levels were measured at 15, 30, 40, 60, 90, and 120 minutes post insulin injection.

Insulin levels were determined in frozen plasma samples obtained from terminally sacrificed mice by ELISA following manufacturer’s instructions (Crystal Chem, Downer’s Grove, IL).

### Treadmill running test

Mice were subjected to exhaustive exercise test on a treadmill as described elsewhere^66^. Briefly, the mice from all groups were initially acclimated to treadmill environment by performing a daily running session at 7 m/min for 15 min during three successive days (adaptation period). Then, the mice run on a treadmill to the point of exhaustion with the following protocol: the starting speed was 9 m/min; the speed was increased 2 m/min every 3 min until it reached 17 m/min; thereafter, the speed continued to increase by 1 m/min every 3 min until exhaustion. Exhaustion was defined as the point at which a mouse refused to run despite being given mild touches.

Blood lactate levels were measured using a lactate assay kit from Abcam following the manufacturer’s instruction. Blood was collected from the tail before and after exercises.

### Isoproterenol-induced cardiac hypertrophy

Isoproterenol was injected peritoneally in mice at 30 mg·kg^−1^day^−1^ for 7 days. Control mice were injected with vehicle only (saline). At day 8, mice were sacrificed. The heart were perfused with saline and dissected into three parts perpendicularly: the apex tip and the aorta proximal part was flash frozen in liquid nitrogen and stored in −80 °C for protein and RNA isolation; the mid section was fixed in formalin for histological study.

### Histological examination of heart tissue

Heart tissues were removed and kept in 10% formalin solution. After routine histologic procedures, they were embedded into paraffin; 4–5 μm thick sections were taken from paraffin-embedded tissues and stained with Picrosirius Red method. Briefly, the paraffin sections were de-waxed and hydrated, followed by staining the nuclei with Weigert’s haematoxylin (Sigma-Aldrich) for 8 minutes, and washing for 10 minutes in running tap water. Then the sections were stained in picro-sirius red (Sigma-Aldrich) for one hour, followed by washing in two changes of acidified water. After dehydrated in three changes of 100% ethanol, the sections were cleared in xylene and mounted in a resinous medium. The stained sections were examined under a Zeiss Axio Lab A1 light microscope (Carl Zeiss, Germany). In general, five heart sections from each mouse out of six mice in each group were examined for fibrosis and collagen content.

### Animal lifespan studies

A total of 67 female mice (27 wild type, 40 *Tsc1* ^*tg*^ mice) and 67 male mice (32 wild type, 35 *Tsc1* ^*tg*^ mice) were used in the lifespan study. Mice were maintained in pathogen-free barrier conditions with 5 mice per cage and were permitted to live out their lives until death due to natural causes with occasional censoring of mice with severe diseases. The survival study was terminated earlier before its completion. The mice that were alive but sacrificed at the termination of the study (18 males on September 16 2014 and 8 females on September 9 2014) were considered censored. Survival data was analyzed using one-sided Log-rank test.

### Statistical analysis

Data were expressed as means ± standard error. Student’s t-test or one-way ANOVA was used to compare between transgenic and wild type mice to determine statistical significances. A p-value of less than 0.05 was considered statistically significant. Survival data was analyzed using one-sided Log-rank test.

## Electronic supplementary material

Supplemental methods and data
