# Supplementary material for: Moderate lifelong overexpression of tuberous sclerosis complex 1 (TSC1) improves health and survival in mice
Source: Sci Rep. 2017 Apr 11;7:834. doi: 10.1038/s41598-017-00970-7 (PMC5429778; doi:10.1038/s41598-017-00970-7)
Supplement: Supplementary file 1 — Supplemental methods and data [file 41598_2017_970_MOESM1_ESM.pdf]

Moderate lifelong overexpression of tuberous sclerosis complex 1 (TSC1) improves health during aging in mice

Hong-Mei Zhang[1*], Vivian Diaz[3], Michael E. Walsh[4,&], Yiqiang Zhang[2,4*]

[1]Department of Oncology, Xijing Hospital, The Fourth Military Medical University, Xi'an, Shanxi, China; [2]Greehey Children's Cancer Research Institute, [4]Department of Cellular and Structural Biology, and [3]Sam and Ann Barshop Institute for Longevity and Aging Studies, The University of Texas Health Science Center at San Antonio, San Antonio, Texas 78229. [&]Current address: ETH Zurich, Department of Health Sciences and Technology, Universitätstrasse 2, 8092 Zurich, Switzerland

Supplemental methods

Respirometry Resting metabolic rate, oxygen consumption, and carbon dioxide production was measured as described elsewhere [9]. Briefly, mice were individually housed with TEK-Fresh cellulose bedding and provided food and water ad libitum during metabolic measurements. The mice were adjusted to the environment for 12 hours prior to the test. The parameters were monitored in a period of 24 hours using a MARS indirect calorimetry system (Sable Systems International, Las Vegas, NV, USA).

Food consumption Food consumption was measured weekly for 4 weeks in a subset of male and female control and TSC1tg mice from the colony (n = 15 per sex and group) at 6 months of age. Mice were single housed during food consumption measurement.

qPCR (quantitative real-time reverse polymerase chain reaction) Total RNA was isolated from tissues using the TRIZOL reagent (Invitrogen), following the manufacturer's instructions. The quality of the RNA was confirmed by agarose gel electrophoresis. After reverse transcribed using the Superscript reverse transcription kit (Ambion), cDNA was used for qPCR using the SYBR green method in an ABI7500 PCR instrument (Life Sciences). GAPDH was used an internal control to normalize the data. Primers used in this study: hTSC1-5F: 5'-CTGGCCAAGAAAGACCACC-3', hTSC1-3R: 5'-CGTCATTACAACAGTCAAGCC-3'.

Grip strength Fore and hind-limb strength was measured using a grip strength meter with mesh grid pull bar (Columbus Instruments 1027 CSM). After allowing the mice to grasp the mesh with both for and hind limbs, mice were pulled horizontally across the grid until grip was lost. For each mouse, 10 consecutive trials were performed and average strength was determined.

Nerve conduction velocity (NCV) and latency All experiments were performed with a Nicolet Viking Quest portable EMG apparatus (CareFusion, San Diego, CA, USA) as described elsewhere [29]. Briefly, mice were anesthetized with isofluorane and maintained at 34 °C with a heating lamp. Subdermal needle electrodes were cleaned with 70% ethanol between animals. Supramaximal stimulation was delivered with 0.02 millisecond electrical impulses for all experiments. Electrodes were inserted 3 cm apart and the latency of the tail distal motor action potential was measured by proximal to distal stimulation. Sciatic NCV was measured by stimulating proximal ankle electrodes with current and the latency for response at the dorsal digits divided by the distance traveled was measured. Then the stimulating electrodes were placed at the sciatic notch and the latency to the ankle was measured, subtracted from the initial food ankle latency and divided by the notch to the ankle distance to obtain values for sciatic NCV.

Passive avoidance test The instrument to conduct passive avoidance test is GEMINI™ system from San Diego Instruments (San Diego, CA). The passive avoidance assay was performed over a period of three days. Briefly, on Day 1 the

mouse was habituated in the right chamber of the apparatus by leaving the mouse in the dark for 30s. After the 30s had passed a light came on in the compartment where the mouse was in and the gate separating the compartments was opened to reveal the dark chamber on the left. The time to move from the lighted to the dark compartment was recorded, with a maximum time limit of 5 minutes. The mouse was immediately taken out of the dark chamber once it crossed over. On Day 2, the mouse was placed in the right chamber as before and left in the dark for 30s. The gate opened after 30s to allow the mouse to move to the dark chamber. Once the animal crossed over a shock (0.2mA) was given for 2s. The mouse was left in the dark chamber for an additional 30s and then removed from the dark chamber. On Day 3, the protocol was the same as for Day 1. Latency to cross over to the dark compartment was recorded, with an upper limit of 5 minutes. Latency to enter an environment associated with an unconditioned aversive stimulus (brief mild electric shock) should be increased if memory of the aversive stimulus is intact.

Echocardiography Mouse cardiac function was examined using echocardiography. Briefly, the mice were put under anesthesia with isoflurane (2.5% isoflurane in 95% O_2 and 5% CO_2). Heart rate was monitored to be within 480-520 beats/min. Anterior and posterior wall thickness and diastolic and systolic left ventricular dimensions will be recorded from M-mode images using method adopted by the American Society of Echocardiography. Fractional shortening is calculated from the end-diastolic diameter (EDD) and end-systolic diameter (ESD) using the equation of (EDD-ESD)/EDD. Estimated echocardiographic left ventricular (LV) mass is calculated as $[(\text{LVEDD} + \text{septal wall thickness} + \text{posterior wall thickness})^3 - \text{LVEDD}^3] \times 1.055$, where 1.055 ($mg/mm^3$) is the density of myocardium.

Animal lifespan studies All mice were fed a standard NIH-31 chow (Teklad Diet LM485; Harlan Teklad, Madison, WI) ad libitum and maintained under barrier conditions in micro-isolator cages on a 12-h dark/light cycle. A total of 47 female (16 WT, 31 $Tsc1^{tg}$) and 43 male (17 WT, 26 $Tsc1^{tg}$) mice were used in the lifespan study. Mice were maintained in pathogen-free barrier conditions with 5 mice per cage and were permitted to live out their lives until death due to natural causes without censoring. The mice used in the lifespan study were not disturbed except to check on the mice twice each day and to remove dead mice. Survival analysis were done using both log-rank (Mantel-Cox) test and Gehan-Breslow-Wilcoxon test. Median survival time was analyzed using Wilcoxon signed rank test.

Table S1. Metabolic and spontaneous activity during light and dark cycles.

Sex	Genotype	Number of mice	LCActivity	LCRQ	LCActivity	DCRQ	RMR
Male	WT	17	0.42965568	0.9087411	0.75217519	0.936219	1.6440263
	TSC1	13	0.44497606	0.8808557	0.78176319	0.914724	1.6120698
	p value		0.46472781	0.2249814	0.44101873	0.260133	0.3988032
Female	WT	18	0.448617	0.847537	0.83825305	0.867755	2.2388151
	TSC1	18	0.45957692	0.903416	0.68967675	0.939996	2.35442
	p value		0.45495836	0.006332	0.17645764	0.002216	0.2244699

The respirometery analysis were performed as described in the methods using mice at 18-20 months of age. Data presented were average value in each group and compared by two-tailed student t-test. Red numbers indicated statistical significance. WT – wild type mice; TSC1 – *Tsc1*^{tg} mice; LCRQ – light cycle respiration quotient (= LCVCO2/LCVO2); LCActivity – light cycle spontaneous activity; DCRQ – dark cycle respiration quotient (= DCVCO2/DCVO2); DCActivity – dark cycle spontaneous activity; RMR – resting metabolic ratio.

Table S2 Echocardiographic data of *Tsc1^{tg}* mouse heart (4 - 6 month of age)

	LV mass (mg)	LVPW;s (mm)	EDD (mm)	ESD (mm)
WT	93.5 ± 7.3	1.2 ± 0.2	3.72 ± 0.18	2.31 ± 0.41
Tsc1^{tg}	92.3 ± 16.3	1.1 ± 0.2	3.83 ± 0.55	2.70 ± 0.91

Male mice at 4-6 months of age were used to conduct the echocardiography as described in the methods. Data presented were average of 6 mice in each group as mean plus standard error. LV mass (mg) was calculated on M-mode and 2-dimensional echocardiography. Values are mean ± SD. LVPW;s – systolic LV posterior wall; EDD – end-diastolic dimension; ESD – end-systolic dimension. n=3

Table S3. Survival data of female and male mice.

Mouse	DOB	Sex	Genotype	DOD	Age (days)	Censoring
TSC1-2113	12/26/10	Female	WT	8/27/12	610	
TSCa-5031	12/1/10	Female	WT	12/18/12	748	
TSCa-5034	12/1/10	Female	WT	12/18/12	748	
TSC1-1026	1/29/11	Female	WT	10/22/12	632	
TSC1-2122	2/16/11	Female	WT	3/1/14	1109	
TSC1-1018	12/4/10	Female	WT	3/26/13	843	
TSC1-2123	2/16/11	Female	WT	7/3/13	868	
TSC1-1029	2/18/11	Female	WT	6/29/13	862	
TSC1-1030	2/18/11	Female	WT	8/14/13	908	
TSC1-1041	3/16/11	Female	WT	3/12/13	727	
TSCa-5068	5/16/11	Female	WT	3/26/13	680	
TSC1-2138	5/22/11	Female	WT	3/26/13	674	
TSC1-2140	5/22/11	Female	WT	3/26/13	674	
TSC1-2164	11/11/11	Female	WT	3/26/13	501	
TSC1-2166	11/11/11	Female	WT	3/26/13	501	
TSC1-2153	10/9/11	Female	WT	3/26/13	534	*
TSC1-2155	10/9/11	Female	WT	3/27/13	535	*
TSC1-2156	10/9/11	Female	WT	2/14/13	494	*
TSCa-5105	11/08/11	Female	WT	3/26/13	504	*
TSC1-1073	10/04/11	Female	WT	12/7/13	795	
TSC1-1075	10/04/11	Female	WT	12/8/13	796	*
TSC1-1066	09/16/11	Female	WT	12/9/13	815	*
TSCa-5107	11/08/11	Female	WT	7/1/14	966	
TSCa-5111	11/16/11	Female	WT	6/14/14	941	
TSC1-1028	2/18/11	Female	WT	2/19/14	1097	
TSC1-1212	06/13/13	Female	WT	9/9/14	453	*
TSC1-1154	01/02/13	Female	WT	2/14/14	408	*
TSC1-1017	12/4/10	Female	Tg	2/28/13	817	
TSC1-2112	12/26/10	Female	Tg	3/12/14	1172	
TSCa-1277	10/7/10	Female	Tg	12/3/12	788	
TSCa-1279	10/7/10	Female	Tg	3/18/13	893	
TSCa-1280	10/7/10	Female	Tg	12/14/12	799	
TSCa-2081	10/5/10	Female	Tg	5/1/12	574	
TSCa-2083	10/5/10	Female	Tg	3/22/13	899	*
TSCa-2084	10/5/10	Female	Tg	8/20/12	685	
TSCa-2085	10/5/10	Female	Tg	3/6/13	883	
TSCa-2086	10/5/10	Female	Tg	3/3/13	880	
TSCa-2087	10/5/10	Female	Tg	3/22/13	899	
TSCa-1284	10/31/10	Female	Tg	3/26/13	877	*
TSCa-1285	10/31/10	Female	Tg	10/15/12	715	*
TSCa-2090	11/17/10	Female	Tg	8/30/12	652	*
TSCa-5036	12/15/10	Female	Tg	11/9/12	695	*
TSCa-2096	12/8/10	Female	Tg	10/1/12	663	*
TSCa-2097	12/8/10	Female	Tg	10/1/12	663	*
TSC1-1024	1/29/11	Female	Tg	6/6/13	859	
TSC1-1045	3/16/11	Female	Tg	10/1/12	565	*
TSC1-2139	5/22/11	Female	Tg	3/26/13	674	*
TSC1-2144	09/06/11	Female	Tg	10/1/12	391	*
TSC1-2149	09/17/11	Female	Tg	3/27/13	557	*

TSC1-1074	10/04/11	Female	Tg	6/5/13	610	
TSC1-2154	10/9/11	Female	Tg	9/9/14	1066	
TSC1-1077	10/26/11	Female	Tg	9/9/14	1049	*
TSC1-1078	10/26/11	Female	Tg	9/12/14	1052	
TSC1-2165	11/11/11	Female	Tg	3/26/13	501	
TSCa-5106	11/08/11	Female	Tg	9/9/14	1036	*
TSC1-1084	11/17/11	Female	Tg	9/9/14	1027	
TSC1-1085	11/17/11	Female	Tg	6/10/13	571	
TSC1-1092	12/09/11	Female	Tg	3/26/13	473	
TSC1-1094	12/30/11	Female	Tg	11/19/12	325	
TSC1-1100	01/01/12	Female	Tg	9/1/14	974	*
TSC1-1101	01/01/12	Female	Tg	9/7/14	980	
TSCa-1153	9/6/09	Female	Tg	10/23/12	1143	
TSCa-2091	11/17/10	Female	Tg	3/26/13	860	*
TSC1-1209	04/22/13	Female	Tg	9/9/14	505	*
TSC1-1210	04/22/13	Female	Tg	9/9/14	505	*
TSC1-1213	06/13/13	Female	Tg	9/9/14	453	*
TSC1-1214	06/13/13	Female	Tg	9/9/14	453	*
TSCa-5100	09/23/11	Male	WT	9/10/13	718	
TSCa-5095	9/2/11	Male	WT	8/28/13	726	
TSC1-1046	4/10/11	Male	WT	3/22/13	712	
TSC1-1055	5/2/11	Male	WT	3/22/13	690	
TSC1-1072	10/04/11	Male	WT	3/22/13	535	
TSC1-2131	4/10/11	Male	WT	9/18/12	527	
TSC1-1048	4/10/11	Male	WT	7/22/13	834	
TSC1-2127	4/10/11	Male	WT	8/26/13	869	
TSC1-2168	12/01/11	Male	WT	10/22/13	691	
TSC1-1070	10/04/11	Male	WT	11/19/13	777	
TSC1-2130	4/10/11	Male	WT	12/12/13	977	
TSC1-2193	02/08/12	Male	WT	12/16/13	677	
TSC1-1088	11/17/11	Male	WT	12/26/13	770	
TSC1-2184	01/16/12	Male	WT	2/18/14	764	
TSC1-1090	12/09/11	Male	WT	2/19/14	803	
TSC1-1079	11/17/11	Male	WT	3/10/14	844	
TSC1-2151	10/9/11	Male	WT	3/17/14	890	
TSC1-2129	4/10/11	Male	WT	4/1/14	1087	
TSC1-2170	12/01/11	Male	WT	4/22/14	873	
TSC1-1128	11/29/12	Male	WT	9/16/14	656	*
TSC1-1129	11/29/12	Male	WT	9/16/14	656	*
TSC1-1132	12/11/12	Male	WT	9/16/14	644	*
TSC1-1133	12/11/12	Male	WT	9/16/14	644	*
TSC1-1134	12/11/12	Male	WT	9/16/14	644	*
TSC1-1141	12/13/12	Male	WT	9/16/14	642	*
TSC1-1195	03/30/13	Male	WT	9/16/14	535	*
TSC1-1187	03/07/13	Male	WT	9/16/14	558	*
TSC1-1173	02/14/13	Male	WT	9/16/14	579	*
TSC1-1174	02/14/13	Male	WT	9/16/14	579	*
TSC1-1147	01/02/13	Male	WT	9/16/14	622	*
TSC1-1206	04/22/13	Male	WT	9/16/14	512	*
TSC1-1071	10/04/11	Male	WT	5/20/14	959	
TSCa-1145	8/19/09	Male	Tg	9/20/10	397	
TSCa-1236	2/4/10	Male	Tg	7/8/11	519	
TSCa-1157	9/12/09	Male	Tg	4/29/11	594	
TSCa-1134	07/10/09	Male	Tg	3/28/11	626	

TSCa-1247	7/14/10	Male	Tg	5/21/12	677	
TSCa-1074	4/16/09	Male	Tg	4/11/11	725	
TSCa-1207	12/12/09	Male	Tg	11/15/11	703	
TSCa-2049	6/21/10	Male	Tg	6/27/12	737	
TSCa-1271	10/7/10	Male	Tg	11/5/12	760	
TSCa-1250	7/14/10	Male	Tg	8/27/12	775	
TSCa-2073	8/25/10	Male	Tg	10/17/12	784	
TSC1-1003	4/4/09	Male	Tg	6/13/11	800	
TSCa-1148	8/19/09	Male	Tg	1/13/12	877	
TSCa-5022	12/3/09	Male	Tg	2/27/12	816	
TSCa-2050	6/21/10	Male	Tg	11/27/12	890	
TSCa-1185	11/14/09	Male	Tg	5/8/12	906	
TSC1-1001	4/4/09	Male	Tg	12/9/11	979	
TSCa-2078	8/25/10	Male	Tg	4/1/13	950	
TSC1-1002	4/4/09	Male	Tg	12/23/11	993	
TSCa-2072	8/25/10	Male	Tg	5/27/13	1006	
TSCa-1184	11/14/09	Male	Tg	10/1/12	1052	
TSC1-2004	7/11/09	Male	Tg	9/14/12	1161	
TSC1-2157	10/19/11	Male	Tg	3/22/13	520	*
TSC1-1076	10/26/11	Male	Tg	3/22/13	513	*
TSCa-1172	10/24/09	Male	Tg	1/6/12	804	
TSCa-1128	6/19/09	Male	Tg	9/23/11	826	
TSC1-1007	5/16/09	Male	Tg	9/7/11	844	
TSCa-2068	8/25/10	Male	Tg	3/14/13	932	
TSC1-1215	09/10/13	Male	Tg	9/17/14	372	*
TSC1-1130	11/29/12	Male	Tg	9/16/14	656	*
TSC1-1131	11/29/12	Male	Tg	9/16/14	656	*
TSC1-1180	02/14/13	Male	Tg	9/16/14	579	*
TSC1-1181	02/14/13	Male	Tg	9/16/14	579	*
TSC1-1182	02/26/13	Male	Tg	9/16/14	567	*
TSC1-1183	03/08/13	Male	Tg	9/16/14	557	*

Supplemental Figures

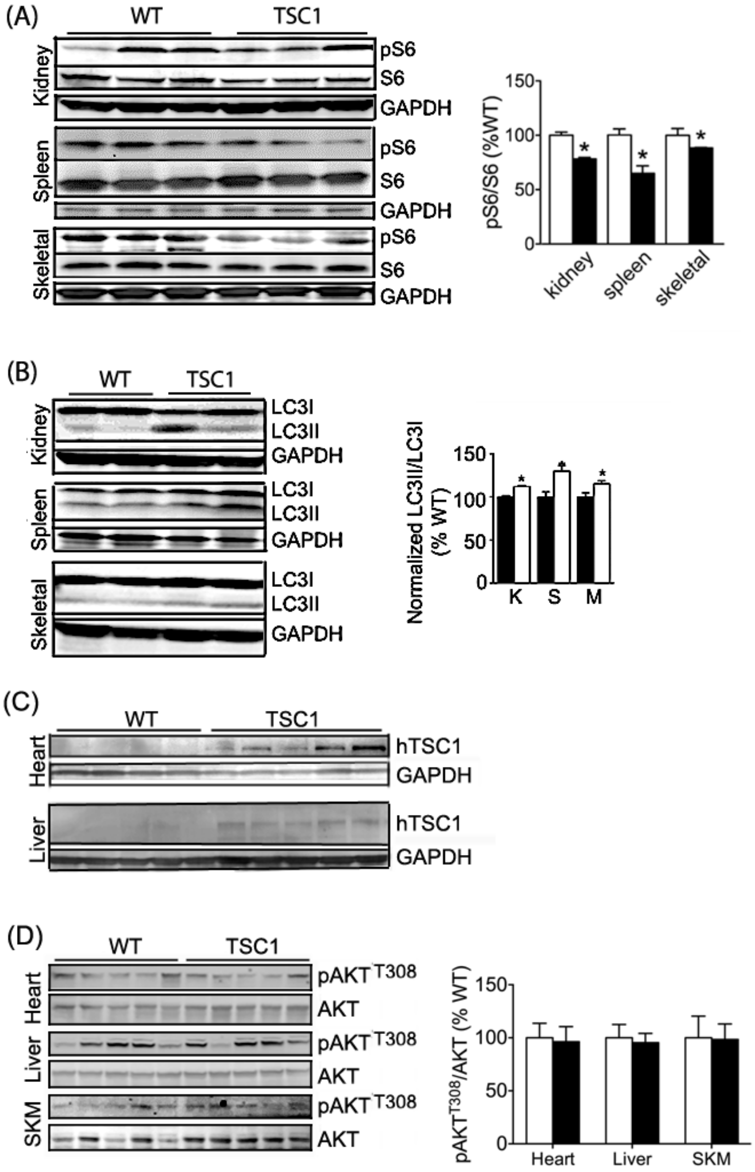

Figure S1. Western blot analysis of mTOR related signaling. Tissue protein extracts from heart, liver and skeletal muscle (SKM) were subjected to Western blotting analysis as described in the material and method section (n = 6 mice for each group). (A) Phosphorylation of S6 in different tissues. (B) Conversion of LC3 in different tissues. (C) hTSC1 protein expression in older mice (24-30 month of age). hTSC1 transgene expression from heart and liver were detected with a human TSC1-specific antibody. (D) Phosphorylation of AKT protein at threonine 308. White bar – wild type mice; black bar – *TSC1*^{tg} mice. Asterisk (*) indicates statistical significance between wild type and *Tsc1*^{tg} mice at $p<0.05$.

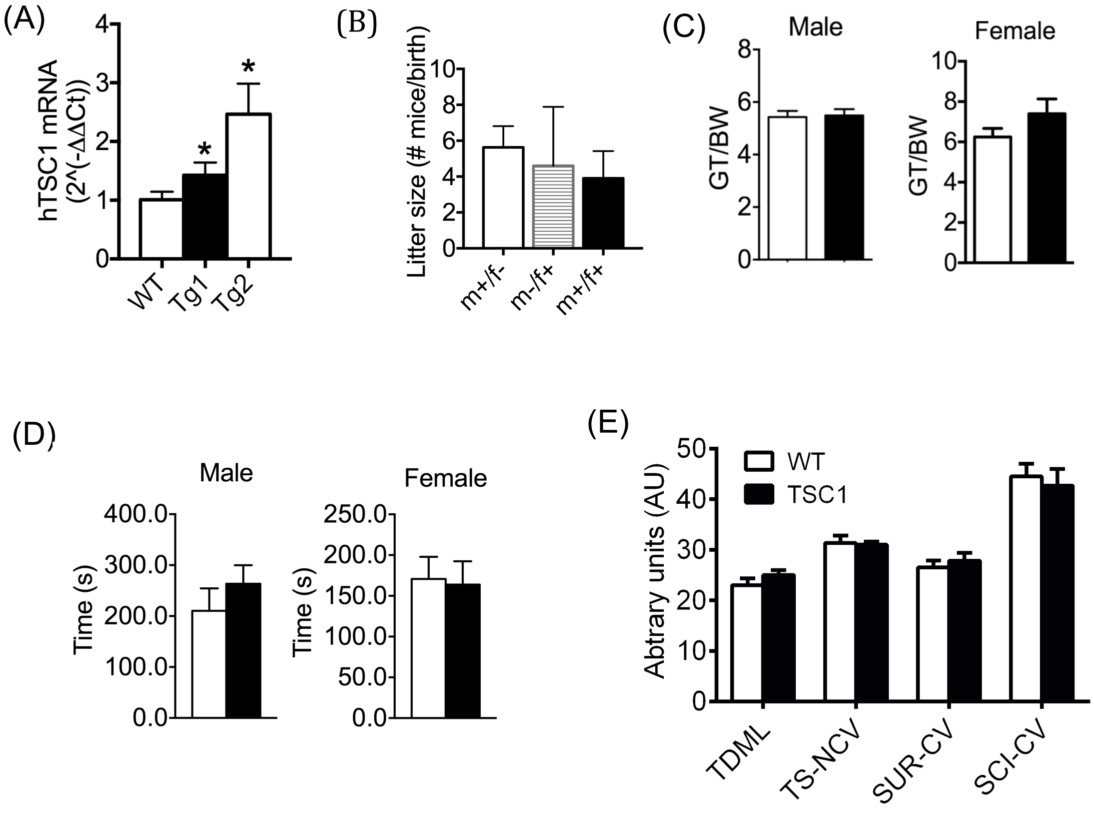

Figure S2. (A) qPCR of hTSC1 transcription. Expression of hTSC1 transgene in neonates (n = 4 in each group) was quantified using qPCR. **(B) Average litter size of different breeding regimes.** m+/f-: male transgenic cross female wild type; m-/f+: male wild type cross female transgenic; m+/f+: male transgenic cross female transgenic. **(C) Grip strength measurement.** $TSC1^{tg}$ mice and age-matched wild type mice (24 – 27 month, n = 8 mice in each group) were subjected to grip strength test as described in methods. **(D) Learning memory comparison between $Tsc1^{tg}$ mice and wild type control mice using the passive avoidance test.** $TSC1^{tg}$ mice and age-matched wild type mice (18 – 20 months of age, n = 10 mice for each group) were subjected to passive avoidance test as described in the methods. The Y-axis represents the time staying in the dark chamber, with the longer the time the worse in memory. White bar – wild type mice; black bar – $TSC1^{tg}$ mice. White bar – wild type mice; black bar – $TSC1^{tg}$ mice. **(E) Functional analysis of nerve conductivity using the nerve conductivity velocity (NCV) test**. Male $TSC1^{tg}$ mice and age-matched wild type mice (24 – 26 month, n = 6 mice in each group) were subjected to nerve conductivity test as described in methods. TS-NCV: tail sensory nerve velocity conductivity; TDML: tail distal motor latency; SUR-CV: sural nerve conductivity velocity; SCI-CV: sciatic nerve conductivity velocity. White bar – wild type mice; black bar – $TSC1^{tg}$ mice.

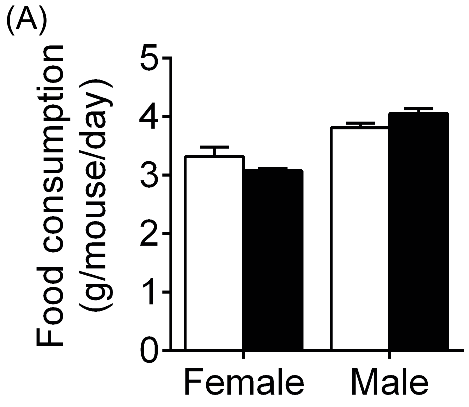

Figure S3. (A) Food consumption. Mice are single housed for 4 weeks (n = 15 in each group). Food consumption was calculated as per gram of food per mouse in each day. White bar – wild type; black bar – *TSC1*^{tg} mice.

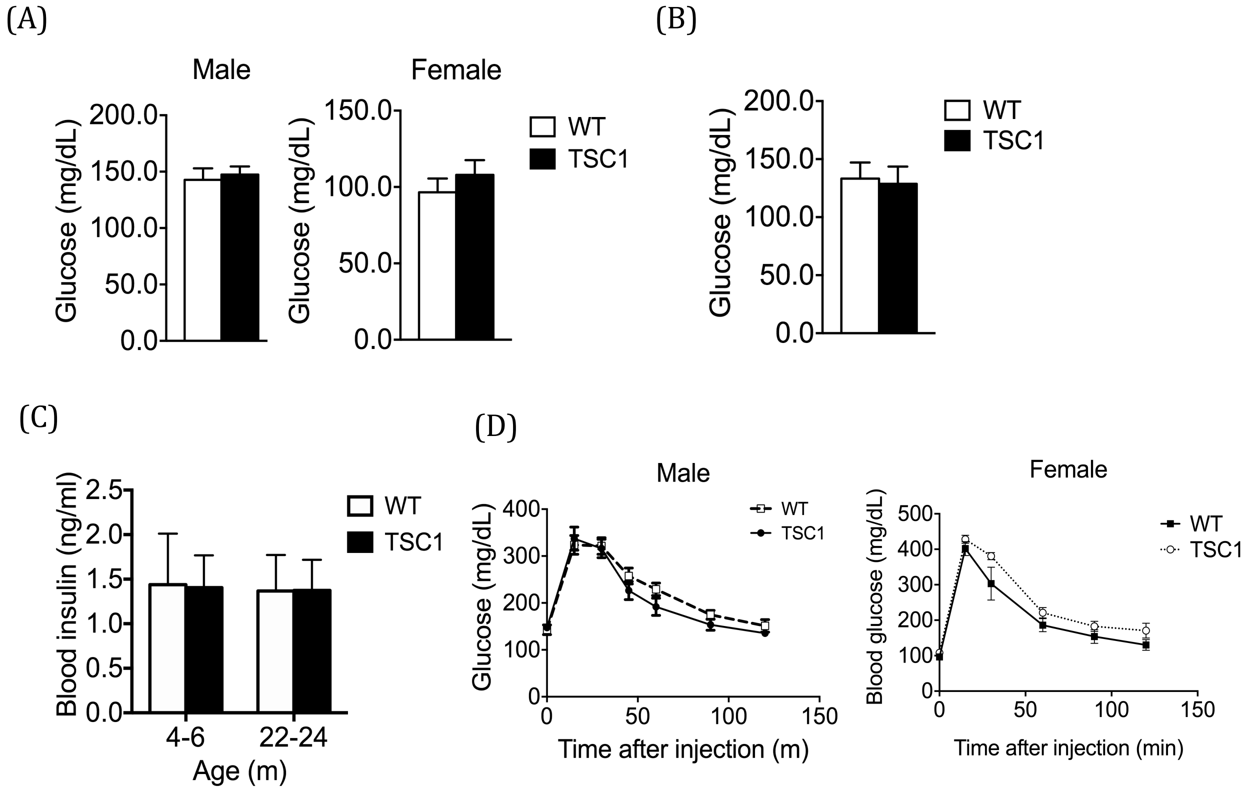

Figure S4. Blood glucose measurement. (A) Fasting blood glucose in 4-6 month old mice overnight (n = 6 in each group). (B) Blood fasting glucose level in 22-24 month old female mice (n = 6 in each group). (C) Blood insulin levels were measured in young and old female mice after terminal bleeding (n = 6 for each group). (D) Glucose tolerance test in younger mice. Mice at 4-6 months of age were subjected to glucose tolerance test as described in the main method section (n = 6 mice in each group). Blood glucose was measured in 20-minutes interval after glucose injection. WT – wild type mice (dotted line or white bar); TSC1 – TSC1 transgenic mice (solid line or black bar).

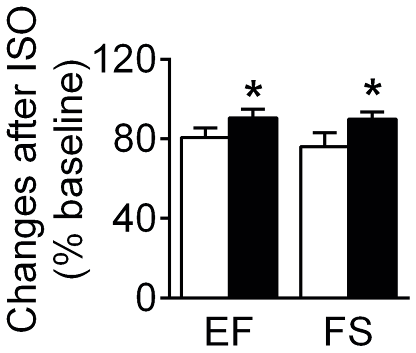

Figure S5. Cardiac function after ISO challenge. 6-8 month old male mice were injected with saline or isoproterenol for two weeks, followed by echocardiography as described in the method section (n = 6). The data were calculated as the changes after ISO challenge against saline injected mice of the same genotype. A value lower than 100% indicates the decrease of cardiac function after ISO challenge. White bar – wild type mice; black bar - *Tsc1*^{tg} mice. EF – ejection friction; FS – friction shortening. Asterisks (*$p<0.05$) indicate statistical significance between wild type and *Tsc1*^{tg} mice.
